# The Effects of Feeding Antibiotic on the Intestinal Microbiota of Weanling Pigs

**DOI:** 10.3389/fvets.2021.601394

**Published:** 2021-03-12

**Authors:** Jeferson M. Lourenco, Rachel S. Hampton, Hannah M. Johnson, Todd R. Callaway, Michael J. Rothrock, Michael J. Azain

**Affiliations:** ^1^Department of Animal and Dairy Science, University of Georgia, Athens, GA, United States; ^2^U.S. National Poultry Research Center, USDA-Agricultural Research Service (ARS), Athens, GA, United States

**Keywords:** antibiotic, bacteria, carbadox, feed efficiency, metabolic pathways, microbiome, microbial diversity, piglet

## Abstract

This study investigated the use of carbadox in the diet of nursery pigs. Ten pens of weanling piglets were assigned to 2 treatments: one containing carbadox and another without it. From days 21 to 35 of age, the first group of piglets was fed carbadox at 55 mg/kg of diet; followed by 27.5 mg/kg from days 36 to 49; and 0 mg/kg from days 50 to 63. The second group of pigs was fed a control diet without carbadox from days 21 to 63 of age. On days 35, 49, and 63, fecal samples were collected directly from the rectum of 2 piglets in each pen, and the samples were subjected to microbial DNA sequencing and metagenomic functional analysis using the 16S rRNA gene. Feed conversion from days 21 to 63 was improved (*P* = 0.04) in the group of piglets fed carbadox. Faith's phylogenetic diversity was similar (*P* = 0.89) for both groups of piglets on day 35, but it was diminished (*P* = 0.01) in the carbadox-fed group on day 49; however, following the complete removal of carbadox from their diets, this microbial diversity index was once again found to be similar (*P* = 0.27) in both groups on day 63. Likewise, abundances of *Slackia, Peptococcus, Catenibacterium, Coprococcus*, and *Blautia* were all similar between the two groups (*P* ≥ 0.40) on day 35, but were smaller in the carbadox group (*P* ≤ 0.05) on day 49; however, on day 63, abundances of all these genera were once again similar (*P* ≥ 0.29). Metabolic pathways involved in cellular growth, death, and genetic information processing (translation) were found to be similarly expressed in the microbiota of piglets from both groups on day 35 (*P* ≥ 0.52), but decreased in the carbadox group on day 49 (*P* ≤ 0.05), and were similar again in both groups on day 63 (*P* ≥ 0.51). These results revealed that feeding carbadox to piglets during the first 4 weeks after weaning significantly affected their fecal microbiotas; however, 2 weeks after the removal of carbadox, those changes tended to disappear, indicating that the shifts were carbadox-dependent.

## Introduction

Antibiotics have given significant contributions to the human food production chain during their almost 80 years of use. Such contributions include a reduced incidence of bacterial disease, improved animal health status, and an overall enhancement in production efficiency (1, 2). However, due to concerns of antimicrobial resistance and its consequences to human health, the entire food-producing industry is under pressure to remove antibiotics from animal production (3). Nevertheless, the complete removal of antibiotics from food-producing systems is not a simple task given that this removal normally results in some loss of productivity and negative economic impacts. In addition, only a limited number of reliable alternatives to antibiotics are currently available (4).

In swine-producing farms, antibiotics are typically used to control diarrhea and improve feed efficiency, which is a trait of utmost importance given that 60–70% of the total cost of production corresponds to feed (5). In US commercial farms, nursery pigs typically gain about 0.667 kg of body weight for each kg of feed consumed [or 1.5 kg of feed for each kg of gain; (6)]. One component that can impact feed efficiency in swine is the bacterial population of their gastrointestinal tracts, as recent studies have shown that pigs with distinct microbiotas have different feed efficiencies (7, 8).

Carbadox is an antibiotic that is widely used in the US swine production, and it is included in feeds to prevent dysentery and improve feed efficiency (9). Currently, the United States Food and Drug Administration (FDA) is evaluating the removal of carbadox from the market. Although this controversial topic is still being debated, carbadox was available in the US market when this study was performed (2019), and it currently is, so there is merit in gaining a better understanding of how this antibiotic works. Furthermore, the exact way by which carbadox improves animal performance is still unclear, and more research is needed to establish how long it takes for the gut microbiota to return to its original state after being exposed to carbadox. Therefore, the present study was performed to evaluate the effects of including carbadox in swine diets during the first two phases after weaning, followed by a removal of this product in a subsequent third phase. To that end, the effects of carbadox on growth performance, feed conversion, and the gastrointestinal microbiota of piglets were assessed during a three-phase nursery rearing system.

## Materials and Methods

### Animals and Treatments

All procedures involving animals performed in this study were approved by the University of Georgia Institutional Animal Care and Use Committee (AUP A2018 01-033-Y1-A0).

Newly-weaned piglets (*n* = 40; 21 days of age; Yorkshire x Duroc crossbred) were obtained from the University of Georgia swine farm and housed in environmentally controlled facilities located at University of Georgia's Large Animal Research Facility in Athens, GA. The piglets were from Choice Genetics (Choice USA, West Des Moines, IA 50266) and were the progeny of the CG36 dam and P26 sire. Upon arrival, piglets were weighed and assigned to 1 of 10 pens, with 4 animals per pen. Each pen (~1.5 × 3 m) hosted 2 males and 2 females and allowed *ad libitum* access to feed and water. Rations for the piglets were formulated to meet National Research Council's recommendations (10) for the duration of the study (i.e., piglets in the nursery stage; 21–63 days-old) and are presented in Supplementary Tables 1–3.

For the first 2 weeks of the nursery period (21–35 days-old), piglets were fed a phase 1 diet. A phase 2 diet was offered from days 36 to 49, and a phase 3 diet from days 50 to 63. Of the 10 pens participating in the study, 5 were randomly assigned to receive a control diet (no antibiotic) and the other 5 were assigned to a diet containing antibiotic. The antibiotic used in this study was carbadox (Mecadox^Ⓡ^, Phibro Animal Health Corporation, Teaneck, NJ). It was included in the phase 1 diet at 55 mg/kg, and at 27.5 mg/kg in the phase 2 diet. No antibiotic was offered to piglets in any of the pens for the last 2 weeks of the study, therefore, all animals in the study were fed the same phase 3 diet—which did not contain carbadox—during the last 14 days of the study (between days 50 and 63). During the entire study, the amount of feed provided, the orts, and the weights of the piglets were assessed on a weekly basis to calculate animal performance and feed efficiency-related parameters.

### Collection of Fecal Samples, DNA Extraction and Analysis

On days 35, 49, and 63 of age, fecal samples were collected from 2 piglets in each pen. The 2 piglets (one male and one female from each pen) were randomly selected on day 35, and subsequently used again on days 49 and 63. The fecal samples were collected by rectal swabs, immediately placed in sterile tubes, and frozen at −20°C. Afterwards, samples were thawed, combined to represent each individual pen, and their microbial DNA was extracted. DNA extractions were performed using the methodology described by Rothrock et al. (11), which uses a combination of mechanical and enzymatic methods to obtain the genomic DNA. Briefly, 0.33 g of fecal material was removed from the surface of the swab and placed into a Lysing Matrix E Tube (MP Biomedicals, Solon, OH), which were homogenized and further processed using an automated procedure. DNA purification was achieved using the DNA Stool–Pathogen detection protocol of the QIAcube Robotic Workstation (Qiagen Inc., Germantown, MD). After purification, DNA concentrations were determined spectrophotometrically using the Synergy H4 Hybrid Microplate Reader (BioTek Instruments, Inc., Winooski, VT). Samples with a minimum volume of 20 μL and 10 ng/μL of DNA were stored at 4°C until the following day. Samples that failed to meet these requirements were rejected and subjected to a new DNA extraction cycle.

One day after the DNA extractions, all samples were taken to the Georgia Genomics and Bioinformatics Core (https://dna.uga.edu) for library preparation and 16S rRNA gene sequencing. PCR libraries were generated using the S-D-Bact-0341-b-S-17 (5′-CCTACGGGNGGCWGCAG-3′) forward and S-D-Bact-0785-a-A-21 (5′-GACTACHVGGGTATCTAATCC-3′) reverse primer pair (12); followed by a PCR clean-up using AMPure XP beads (Beckman Coulter Life Sciences, Indianapolis, IN, USA). Libraries were quantified using qPCR, and nucleotides were then sequenced using an Illumina MiSeq instrument and a MiSeq v3 reagent kit (Illumina Inc., San Diego, CA, USA). A bacteriophage PhiX genome (PhiX Control v3 Library; Illumina Inc., San Diego, CA, USA) was used as a control for the sequencing runs.

Sequencing data were demultiplexed and converted into FASTQ files. Paired-end sequencing reads were imported into the software Geneious v11.1.5 (Biomatters Ltd., Auckland, New Zealand) and then merged. Merged files were exported from Geneious as individual FASTQ files and were quality-filtered according to the default values provided in the “multiple_split_libraries_fastq.py” script in the QIIME pipeline v1.9.1 (13). The files were then converted into the FASTA format, and sequences were clustered into operational taxonomic units (OTU) at 97% similarity using the Uclust OTU picking method. Samples that did not align to PyNAST were excluded from the analysis. The sequencing depth was set at 33,200 sequences per sample for further analysis. Bacterial groups that had relative abundances smaller than 0.1% were shown as parts per million (PPM). Phylogenetic Investigation of Communities by Reconstruction of Unobserved States (PICRUSt) was carried out to make inferences about the metabolic functions of the microbial community (14, 15). Metagenome metabolic functions were assessed using the Kyoto Encyclopedia of Genes and Genomes (KEGG) second-level pathways.

### Data Availability and Statistical Analysis

Nucleotide sequencing data was deposited in a public repository (www.mg-rast.org) under accession number mgm4906046.3. Statistical analyses were performed using the software Minitab® v18.1 and R (v3.3.3). Data were analyzed by ANOVA for each individual day (i.e., ages of 21, 35, 49, and 63 days-old) using the two groups—Antibiotic or No Antibiotic—as factors, and pens were considered the experimental unit. Average daily feed intake was evaluated using linear regression to assess the progression of feed intake over time. All results were treated as trends when *P* ≤ 0.10, and were declared statistically significant when *P* ≤ 0.05.

## Results

### Animal Performance

**Figure 1:**
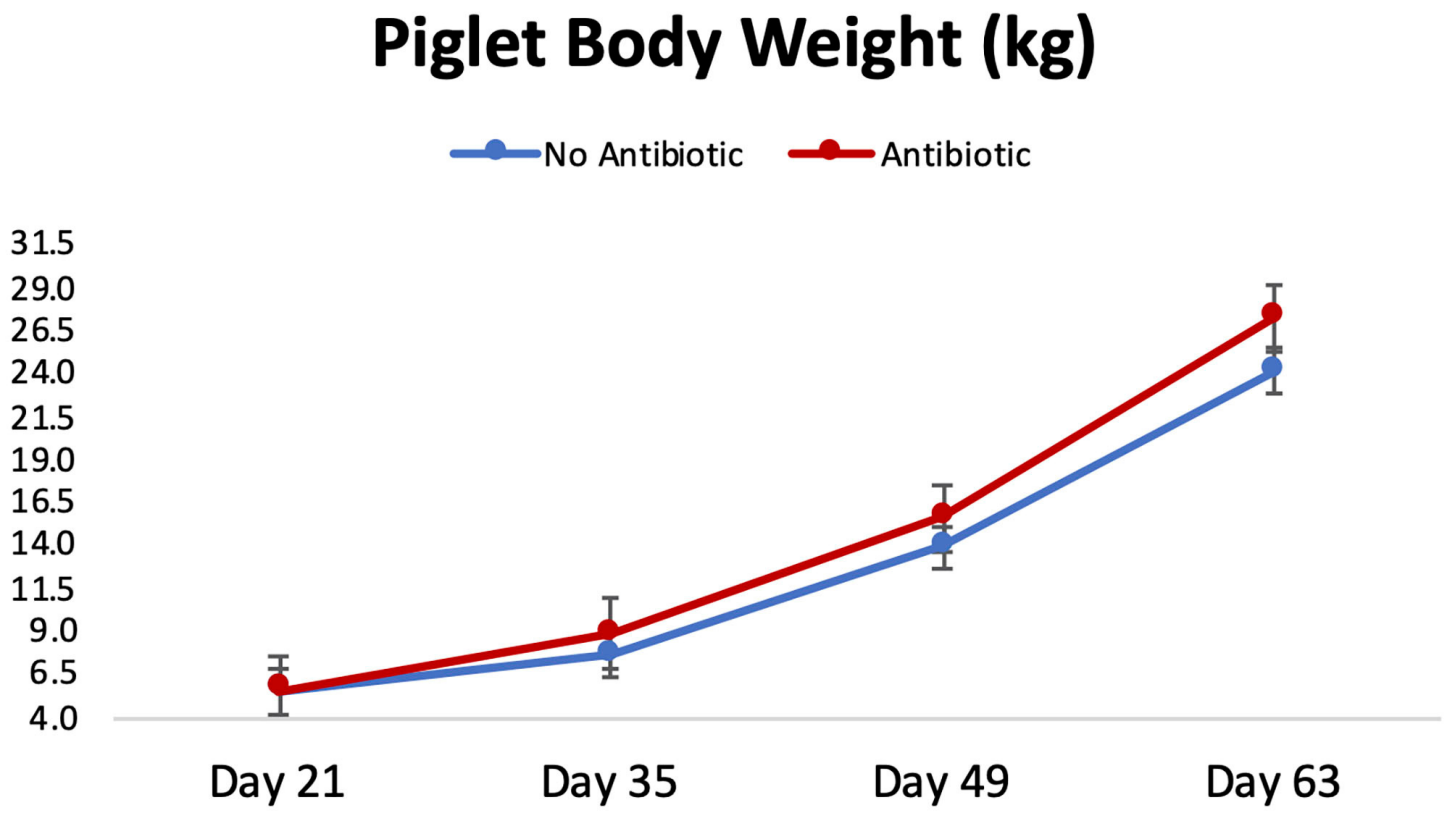
Body weight progression observed for piglets in the No Antibiotic and Antibiotic groups during the study. No significant differences were observed between groups for all the days shown (*P* ≥ 0.46).

**Figure 2:**
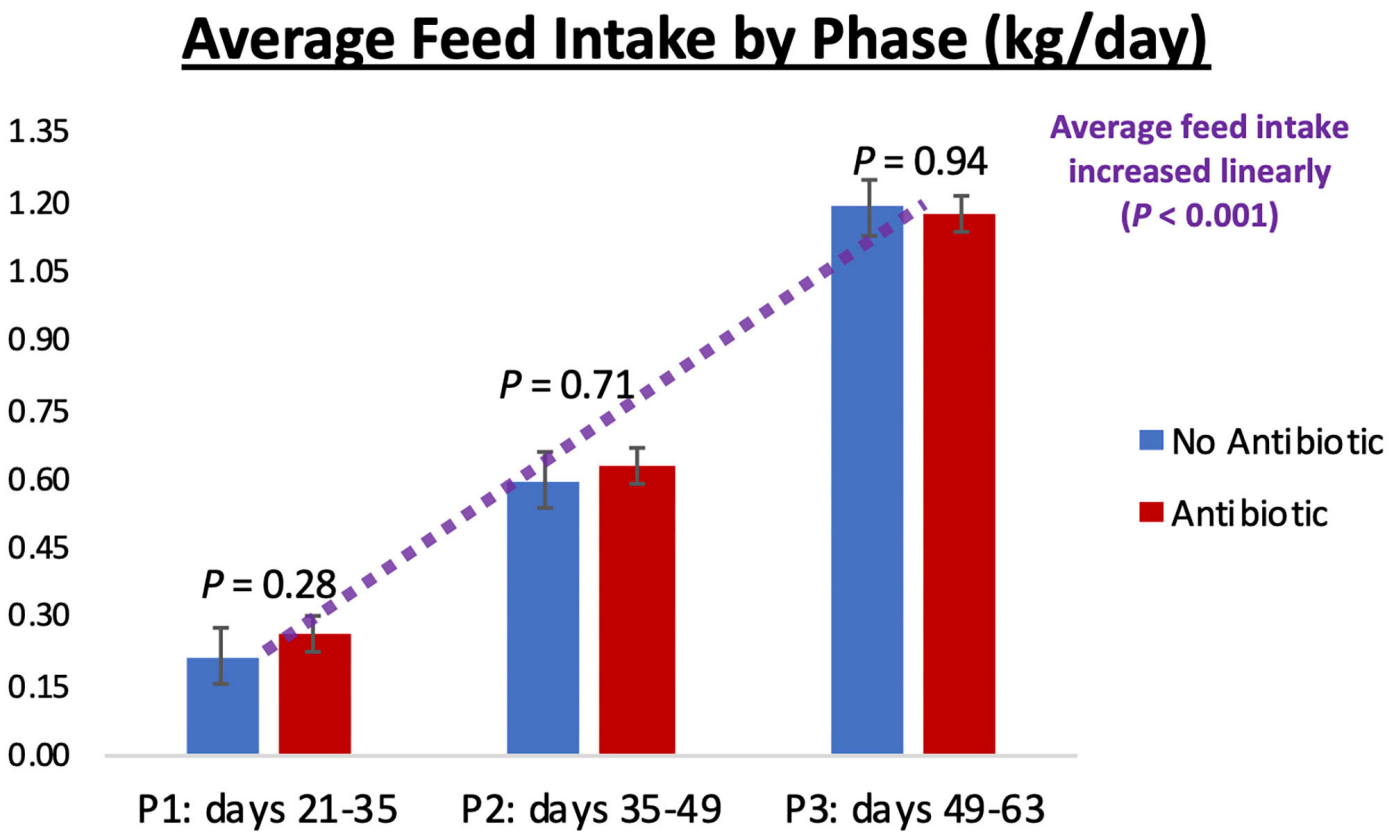
Average feed intake observed in phases 1, 2, and 3 of the study (21–35; 35–49; and 49–63 days-old, respectively). No differences (*P* ≥ 0.28) were observed between the No Antibiotic and Antibiotic groups, but feed intake linearly increased (*P* < 0.001) as piglets went from phase 1 to phase 3. Average intake for phases 1, 2, and 3 were 0.24, 0.61, and 1.18 kg/day, respectively.

**Figure 3:**
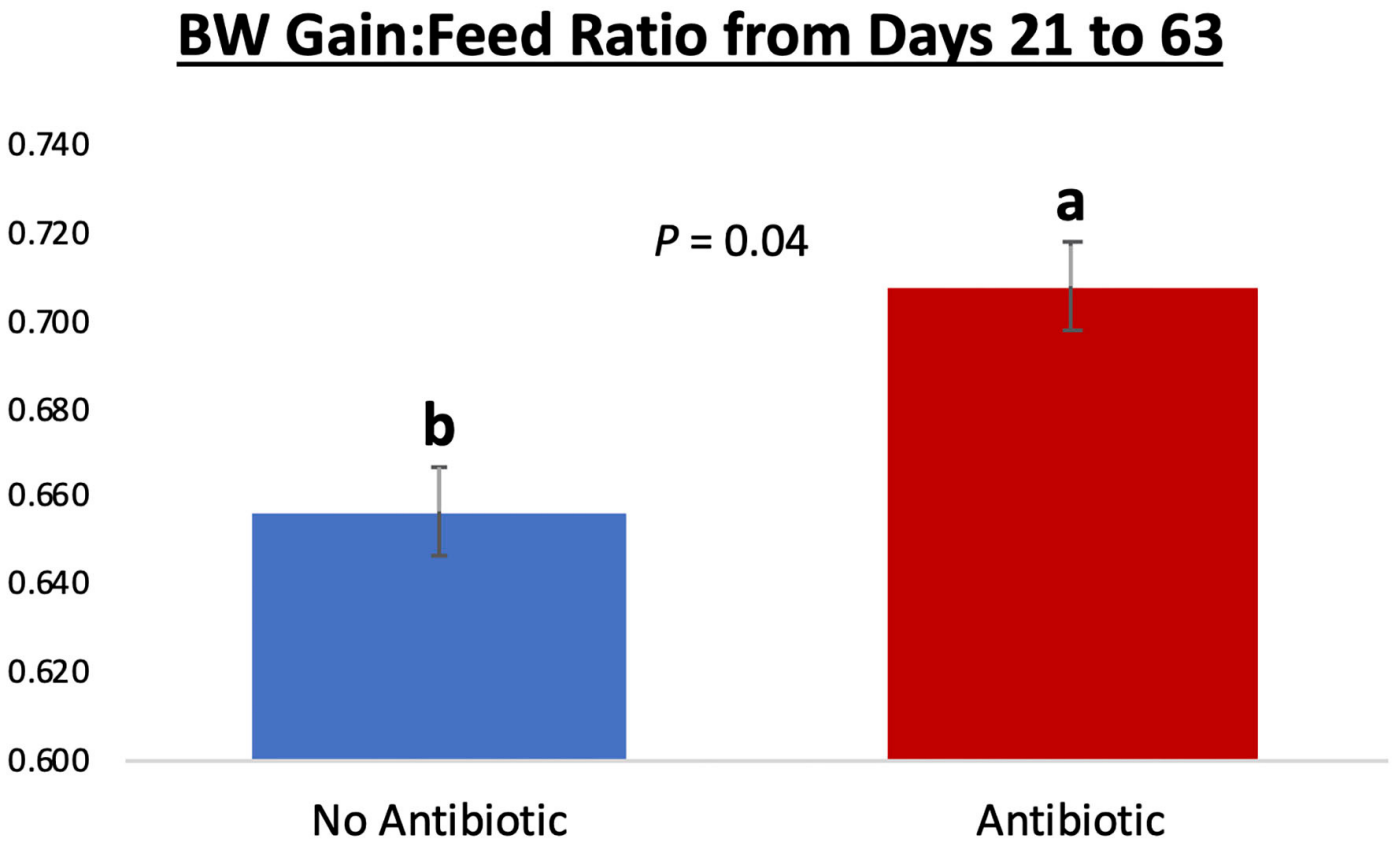
Feed efficiency (expressed as gain: feed ratio) for piglets in the No Antibiotic and Antibiotic groups observed over the course of the entire study. Feed efficiency was significantly improved in the Antibiotic, compared to the No Antibiotic group (0.7075 vs. 0.6564; *P* = 0.04; which is equivalent to 1.41 and 1.52 kg feed:1 kg body weight gain, respectively). No antibiotic was given in the phase 3 of the study.

Piglet body weight was similar at the beginning of the study (day 21; Figure 1); however, as the study progressed, piglets in pens treated with antibiotics had numerical greater body weights compared to the ones not receiving it, but this numerical increase was not statistically significant for any of the days evaluated (*P* ≥ 0.46). Similarly, in Figure 2, it can be seen that there were no differences (*P* ≥ 0.28) in average feed intake between piglets in the two treatment groups. But when comparing the three phases of the study, feed intake linearly increased (*P* < 0.001) in both groups as the piglets got older. Although no antibiotic was offered during the last 14 days of the study (phase 3), feed efficiency was calculated for the entire course of study and is shown in Figure 3. Significant differences (*P* = 0.04) were found between the two treatment groups, with piglets that received antibiotics being more efficient than the ones that never received antibiotics (gain:feed = 0.7075 and 0.6564, respectively).

### Changes in the Microbiota

**Figure 4:**
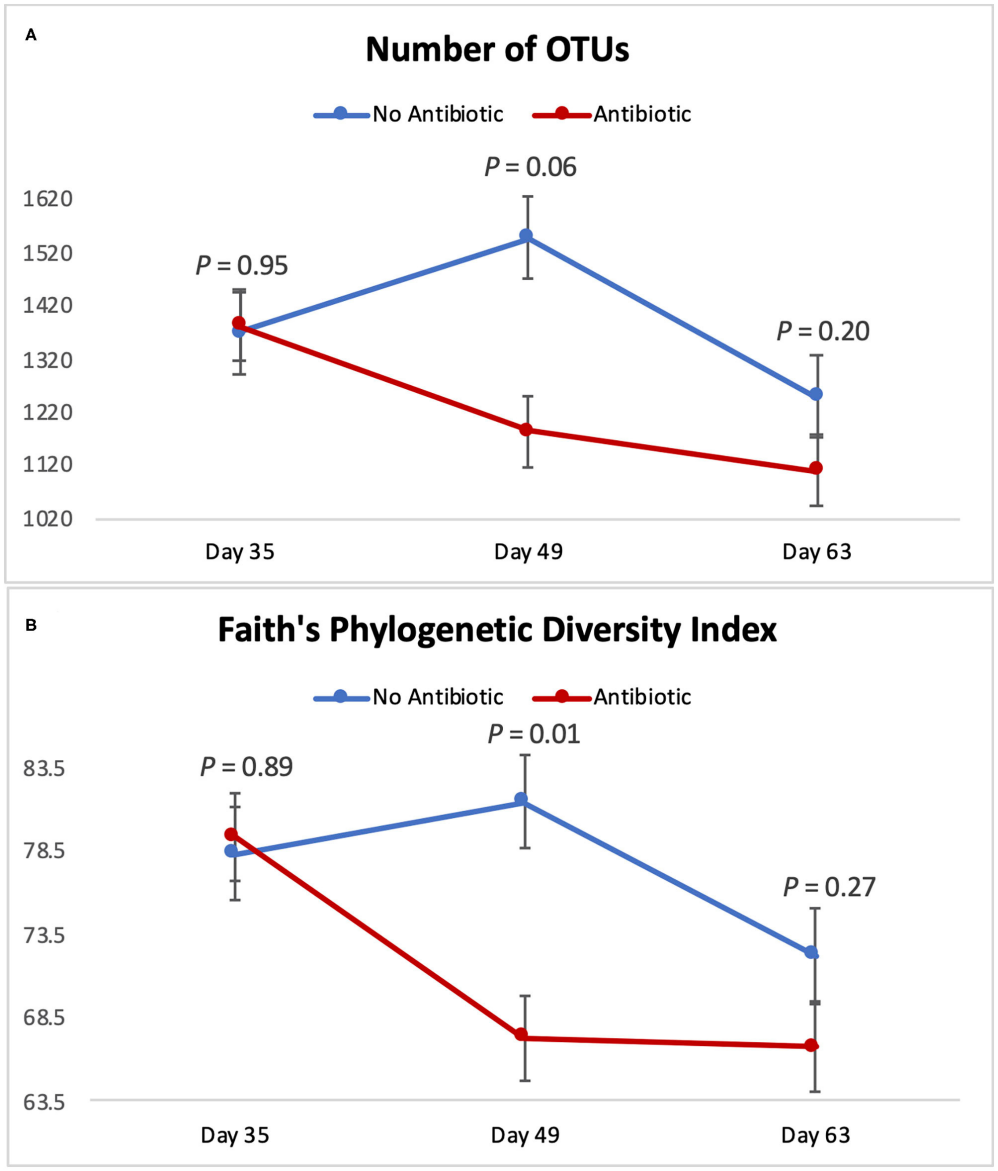
Indicators of microbial richness and diversity for piglets in the No Antibiotic and Antibiotic groups: **(A)** Number of OTUs (richness indicator); **(B)** Faith's Phylogenetic Diversity Index (diversity indicator). *P*-values indicate the contrast between piglets in the two groups on each (day 35: end of phase 1; day 49: end of phase 2; day 63: end of phase 3).

Figure 4 summarizes microbial richness (the number of OTUs) and microbial diversity (Faith's phylogenetic diversity) of the piglet gut microbiomes. The overall behavior of those two indexes was similar throughout the study: No differences were observed on day 35 (*P* ≥ 0.89); but on day 49, Faith's phylogenetic diversity was significantly decreased (*P* = 0.01) in the group fed antibiotic. Likewise, the number of observed OTUs tended to be lower (*P* = 0.06) on day 49 in piglets fed antibiotic. However, on day 63, no differences were detected (*P* ≥ 0.20) between piglets from the two groups.

**Figure 5:**
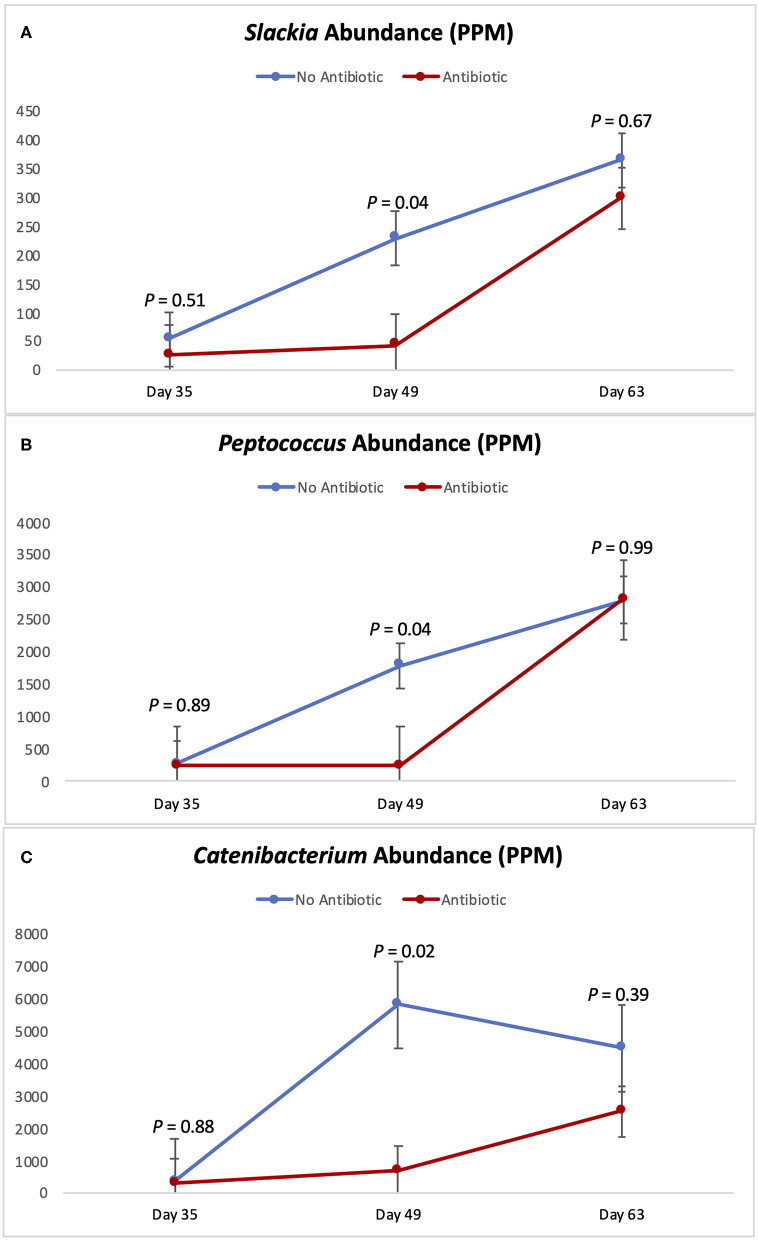
Abundance of the genera *Slackia* **(A)** *Peptococcus* **(B)** and *Catenibacterium* **(C)** observed in the feces of piglets in the No Antibiotic and Antibiotic groups. *P*-values indicate the contrast between piglets in the two groups on each (day 35: end of phase 1; day 49: end of phase 2; day 63: end of phase 3).

**Figure 6:**
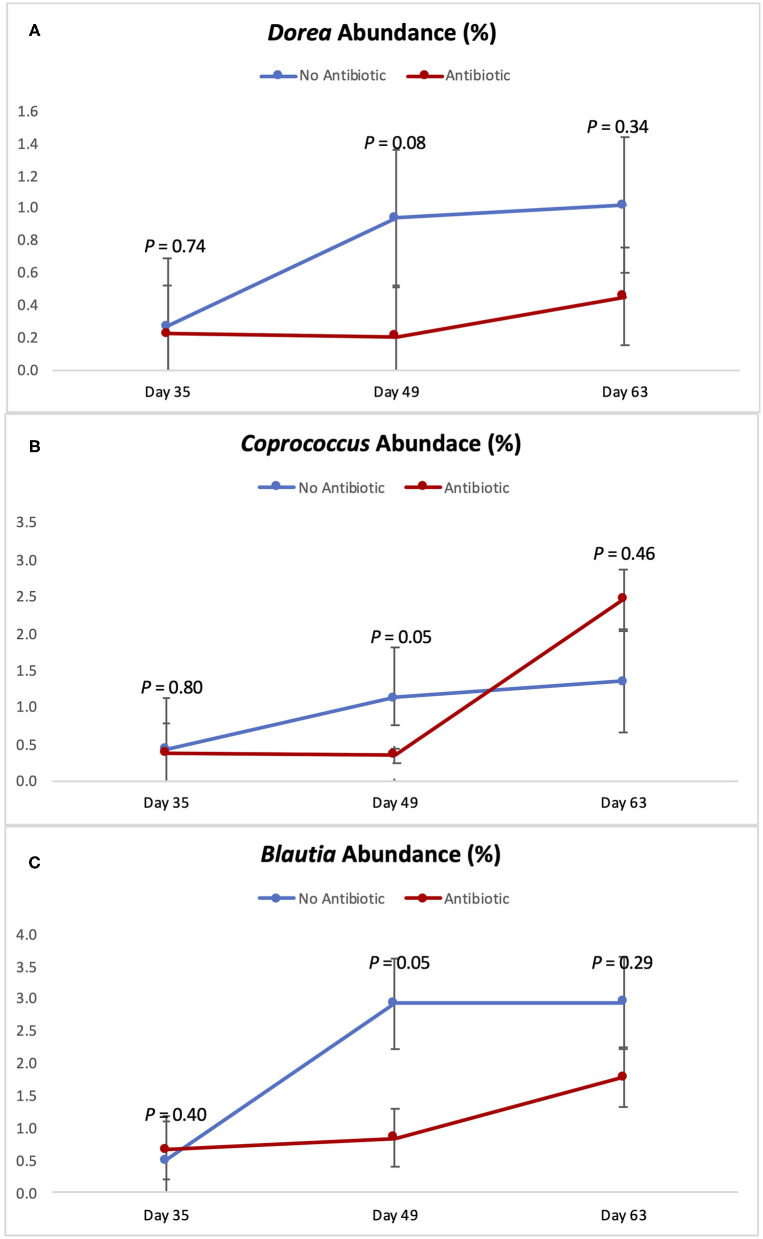
Abundance of the genera *Dorea* **(A)** *Coprococcus* **(B)** and *Blautia* **(C)** observed in the feces of piglets in the No Antibiotic and Antibiotic groups. *P*-values indicate the contrast between piglets in the two groups on each (day 35: end of phase 1; day 49: end of phase 2; day 63: end of phase 3).

Figures 5, 6 show the changes in abundance observed in specific bacterial genera during the course of the study. While all the bacteria shown there had the same abundance on day 35 (*P* ≥ 0.40), significant decreases (*P* < 0.05) in the populations of *Slackia, Peptococcus, Catenibacterium, Coprococcus*, and *Blautia* were observed in the group fed antibiotics by day 49, as well as a tendency for smaller abundance of *Dorea* (*P* = 0.08). On day 63 however, the abundances of all those bacterial genera were found to be similar again (*P* ≥ 0.29).

### Changes in Expression of Metabolic Pathways

**Figure 7:**
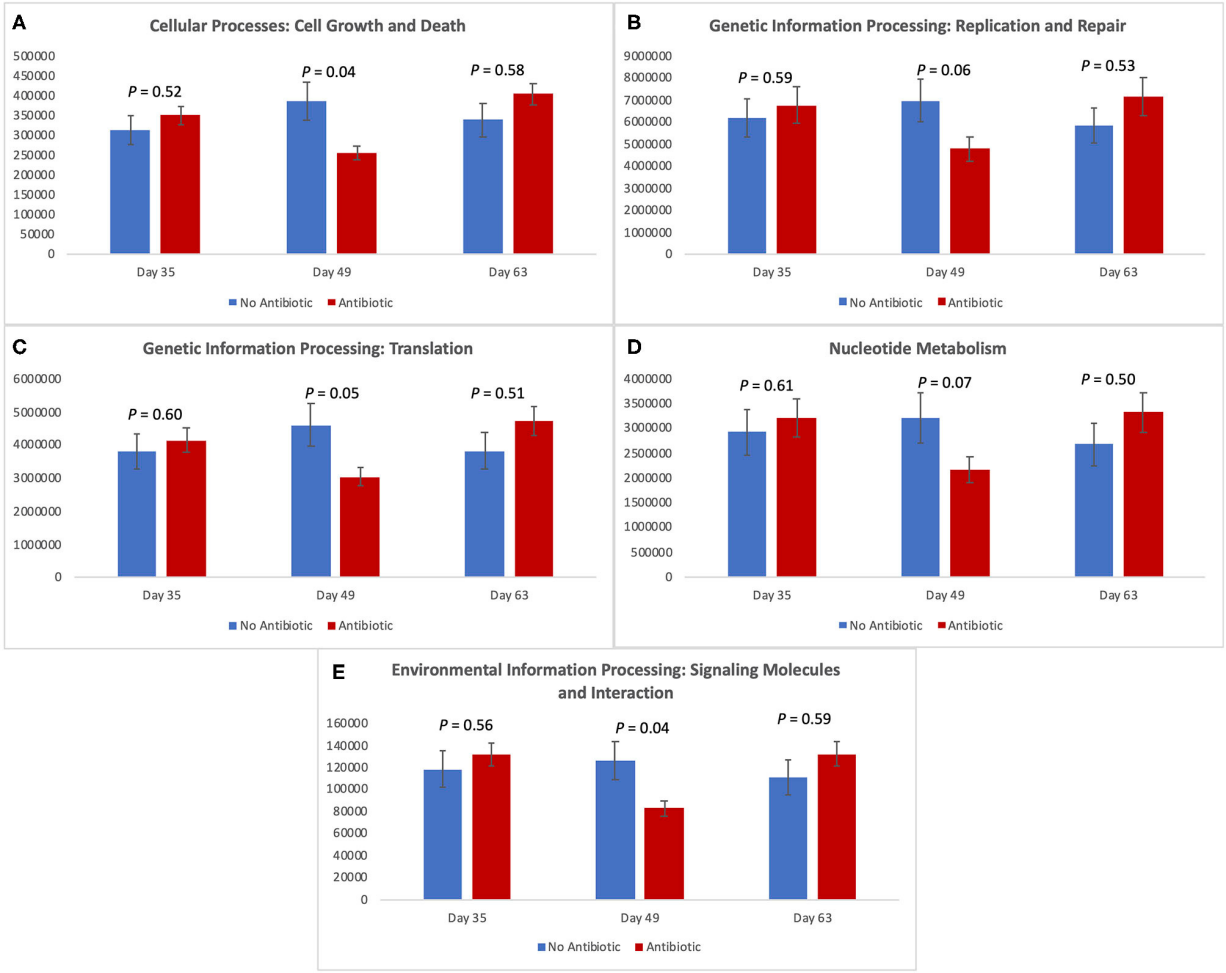
Expression of level-2-KEGG pathways in the fecal microbiota of piglets in the No Antibiotic and Antibiotic groups: **(A)** Cell growth and death; **(B)** Genetic information replication and repair; **(C)** Translation; **(D)** Nucleotide metabolism; **(E)** Signaling molecules and interaction. *P*-values indicate the contrast between piglets in the two groups on each (day 35: end of phase 1; day 49: end of phase 2; day 63: end of phase 3).

Figure 7 shows the expression of five metabolic pathways from the gut microbiomes of the antibiotic and antibiotic-free piglets throughout the study. The five level-2 KEGG pathways are related with cellular growth and death, genetic information and processing, nucleotide metabolism, and environmental information processing. For all of them, there was no difference in expression on day 35 (*P* ≥ 0.52); but as was observed with the other microbiome data, significant (*P* < 0.05) or trending toward significant (*P* < 0.07) changes were observed for all pathways on day 49. However, once again, no differences were observed on day 63 (*P* ≥ 0.50) between the two piglet treatment groups.

## Discussion

### Effects of Carbadox on Performance of Piglets

Carbadox has been used as a feed additive in diets of young pigs for over 50 years, and its use is well-documented (9, 16–18). In the current study, carbadox was fed at 55 mg/kg in phase 1 (21–35 days old) and 27.5 mg/kg in phase 2 (36–49 days old) in the ration of the piglets that were treated. Thrasher et al. (16) conducted several growth trials using weanling pigs and tested inclusion levels of carbadox ranging from 18.3 to 110 mg/kg of feed. The authors found that the greatest improvements in weight gains were obtained by including it in the range of 27.5–55 mg/kg of feed. In addition, authors reported that the average daily gain in piglets suffering from diarrhea was 60% greater when carbadox was present at 27.5 mg/kg, compared to the control group (i.e., no antibiotics). Moreover, authors observed improved feed efficiency when carbadox was included in the diets of piglets. Differently than what was reported by Thrasher et al. (16), the current study did not detect differences in average daily gain for piglets receiving carbadox; however, we observed a significant improvement in feed efficiency for the group of pigs that received carbadox during phases 1 and 2.

Yen et al. (17) performed five feeding trials using carbadox, and all of them had this antibiotic added at 55 mg/kg of feed. Two of those trials were conducted with older pigs (initial age: 10–11 weeks old), but the other three trials were conducted using 4–5 week-old pigs, which are comparable to the present study. In two of those three trials, the authors did not find differences in animal performance, but in one of them, they reported significant improvements in average daily gain, feed efficiency, and also an increased daily feed intake due to inclusion of carbadox. Harper et al. (18) also tested the inclusion of 55 mg of carbadox per kg of diet in two trials using crossbred weanling pigs and found positive results. In both of their trials, authors observed increased average daily gains in the first 5 weeks post-weaning, and in their second trial, inclusion of carbadox improved feed efficiency in the first 3 and in the first 5 weeks post-weaning, which is in line with our findings.

### Changes in Piglet's Microbiota During the Study

As can be seen in Figures 4–6, when the piglets were on average 35 days-old, the fecal microbiotas from both groups was very similar. In fact, except for some numerical differences, no significant dissimilarities were observed when comparing the antibiotic to the no antibiotic group. At that age, piglets had been fed the phase 1 diet for 2 weeks, and the level of carbadox in the diet of the antibiotic-fed group was 55 mg/kg; however, as shown in Figure 2, feed intake during phase 1 was very low compared to the following two phases, since the piglets consumed on average only 240 g feed/day during this initial phase. Thus, although the level of carbadox in the antibiotic group was the highest in this study during phase 1, the amount of feed piglets consumed in that period was likely not enough to generate important shifts in their microbiomes. However, feed intake linearly increased in both groups as the study progressed (Figure 2). Consequently, compared to phase 1, feed intake more than doubled during phase 2 as pigs consumed an average of 610 g of feed per day. Despite the lower level of carbadox used in the antibiotic group during phase 2 (i.e., 27.5 mg/kg), the greater intake of feed experienced by the piglets led them to consume greater amounts of antibiotics daily. This greater intake likely played a major role in generating the distinctions in the microbiotas of the piglets that were observed on day 49, particularly the reduction in microbial richness and diversity in the antibiotic-fed group. Another possible explanation for the observed differences is that it may take some time for the cumulative build-up effects of carbadox on the microbiome to take place. Nevertheless, despite the differences seen on day 49, at the end of phase 3 (i.e., day 63), when both groups of pigs were fed the same diet—which did not have carbadox included—no significant differences in their microbiotas were observed. This lack of differences indicates that feeding piglets the same diet for a period of 2 weeks is likely enough time to equalize their gastrointestinal microbiomes, despite the existence of previous differences.

Microbial richness and diversity followed a similar pattern during the 3 phases of the study: While no differences were detected on day 35, significant alterations were observed 2 weeks later, with the antibiotics group having a lower diversity and a tendency to have lower richness compared to the control group (Figure 4). In line with our findings (9), reported that feeding piglets a ration with 50 mg of carbadox per kg of feed significantly decreased their intestinal microbial richness (number of OTUs) compared to a control group; however, they observed this effect earlier than we did: While those authors saw a reduction in the number of OTUs in the 1st week after the inclusion of carbadox in the piglets' diet, in the present study, we observed this effect only on day 49, when carbadox had already been fed for 4 weeks. Despite this distinction between the two studies concerning the number of days it took to observe changes in piglet's microbiomes, both (9) and the present study found that the removal of carbadox from the diet resulted in an equalization in the number of OTUs in the feces of piglets, indicating a short-term nature of the effects of carbadox.

Microbial diversity, expressed as Faith's phylogenetic diversity, was also found to be lower in the antibiotic-fed group on day 49; but once again, this difference in diversity was gone by day 63, when carbadox had been removed from the feed. Once again, this shows that feeding the common phase 3 diet for the last 2 weeks of the study was enough to eliminate differences in the gastrointestinal microbiomes of the piglets. A previous study from our group (19) observed a similar effect on alpha-diversity in cattle. In that study, commingling and feeding weanling cattle the same diet for a period of 4 weeks completely eliminated the initial differences in alpha-diversity existing in their ruminal microbiotas (i.e., Shannon diversity index and Faith's phylogenetic diversity). Similarly (9), reported a reduction of 31.3% in the number of OTUs in piglets that received carbadox compared to a control group that did not receive this antibiotic, and this difference disappeared after the removal of carbadox from the piglets' diet. Therefore, our results are in line with the ones previously observed in the literature, given that feeding the piglets the same diet for 2 weeks resulted in the equalization of their gastrointestinal microbial diversity, regardless of the previous differences.

According to Riviere and Papich (20), carbadox is an antibacterial agent that is primarily active against gram-positive bacteria. Thus, not surprisingly, at the end of phase 2 (day 49), the presence of carbadox in the diet had significantly decreased the populations of *Slackia, Peptococcus, Catenibacterium, Dorea, Coprococcus*, and *Blautia*, which are all gram-positive bacteria. However, interestingly, the 14-day withdrawal period that took place when piglets were between 49 and 63 days-old was enough time to re-establish the abundance of all those bacterial genera to the same levels of the no antibiotic group. This indicates that these bacterial groups have great plasticity, and that a 14-day removal is enough time for them to have their populations re-established.

In general, antibiotics cause a decrease in gut microbial diversity (21); however, they normally induce piglets to greater rates of body weight gain and feed efficiency (18, 22). Although this seems counterintuitive, it has been established that more efficient microbiomes are not necessarily more diverse (23). In fact (23), have found that more efficient microbiomes can be less diverse and produce a smaller range of output metabolites; however, in that smaller pool of metabolites there can be a larger amount of biologically relevant metabolites, which are more readily available to be utilized by the host animal. Similarly (24), found that nursing calves that received supplementation in their diets had greater amounts relevant metabolites (i.e., short chain fatty acids; which can readily be utilized by the animal for energy), which resulted in a numerically greater body weight gain, despite the fact that the gut microbiota of supplemented calves had lower diversity. Likewise, it has been demonstrated that pigs receiving antibiotics had increased expression of functional genes of their microbiota related to energy production and conversion, indicating that the consumption of antibiotics results in a more efficient capture of energy from the feed (25). Therefore, less diverse microbiomes can in fact be more efficient than the ones with greater diversity due to the quality of the metabolites that are generated, and this fact can explain why animals that received carbadox in this study had better feed conversion despite having a less diverse intestinal microbiota.

### Metagenomic Predictions

As revealed by the analysis of the level-2-KEGG metabolic pathways, overall, the pathways involved in cellular processes such as cell growth and death, and processing of genetic and environmental information were all affected, or tended to be affected on day 49, despite the lack of differences observed earlier (i.e., day 35). Similar to what was observed in the overall microbial diversity, the constant presence of carbadox for 28 days resulted in a significant decrease in the expression of the studied pathways in the intestinal environment of antibiotic-fed pigs. However, 14 days after the removal of carbadox from the diets of the pigs (i.e., day 63), all the metabolic pathways returned to similar levels of expression in both treatment groups. These results indicate that the gut microbiota of young pigs have a great degree of malleability. In addition, the gene expressions and metabolism of such microbiota also have this attribute.

According to Constable et al. (26), the exact mechanism of action by which carbadox kills bacteria (primarily gram-positive) is not known. In spite of that, it is known that antibiotics act by disrupting essential processes in the bacterial cells, which impair some bacteria and kill others. Life within a cell requires a delicate balance between the promotion and inhibition of growth (27). Thus, although its specific mode of action is not completely elucidated, by day 49 carbadox was able to decrease the number of genes involved in cellular processes controlling growth and death. In addition, the processing of genetic information (translation) and the processing of environmental information were also affected by the presence of carbadox. These results not only confirm, but they also complement, the ones obtained by evaluating the piglet's microbiota; however, they are not totally unexpected given that the metagenomes were predicted using the same sequencing data used for the taxonomic analysis. In spite of that, our results indicate the potential mechanisms and pathways that carbadox tend to inhibit.

## Conclusion

Overall, although performed with a relatively small sample size, our study has consistently shown that the gut microbiota of weanling pigs have a great degree of malleability. When the antibiotic carbadox was present in their diets for a total of 28 days after weaning, important changes were observed in their intestinal microbiotas, both at the genus level and in the overall microbial richness and diversity. In addition, the metabolic pathways expressed by their intestinal microbiotas were also affected by the presence of carbadox. However, after a complete removal of carbadox from their diets for 14 days (during phase 3), virtually all of the observed differences disappeared, indicating the ability that the intestinal microbiota of piglets has to return to its normal state in a relatively small amount of time. These results indicate the potential for short-term applications of carbadox, which may benefit piglets in terms of reduction of diarrhea and increase of feed efficiency, while not affecting piglet gut microbiomes. But if a longer term treatment (≥4 weeks) is needed, up to 2 weeks is needed for the gut microbiome to return to normal after the end of the antibiotic treatment. No further information regarding the health and physiology of the pigs were evaluated, so further studies dealing with these topics should be performed given the close connection between microbiota and host health.

## Data Availability Statement

The datasets presented in this study can be found in online repositories. The names of the repository/repositories and accession number(s) can be found in the article/Supplementary Material.

## Ethics Statement

The animal study was reviewed and approved by University of Georgia Institutional Animal Care and Use Committee.

## Author Contributions

MA, TC, and JL were responsible for the conception of the study. MA, RH, and JL were responsible for acquisition of data. JL was responsible for the statistical and data analysis. MR, JL, and RH were responsible for laboratory work. MA, RH, HJ, MR, TC, and JL were responsible for writing the manuscript. MA supervised the collection of data and manuscript editing. All authors have read and agreed to the published version of the manuscript.

## Conflict of Interest

The authors declare that the research was conducted in the absence of any commercial or financial relationships that could be construed as a potential conflict of interest.
