# Supplementary material for: The Effects of Feeding Antibiotic on the Intestinal Microbiota of Weanling Pigs
Source: Front Vet Sci. 2021 Mar 12;8:601394. doi: 10.3389/fvets.2021.601394 (PMC7996051; doi:10.3389/fvets.2021.601394)
Supplement: Supplementary file 1 [file Data_Sheet_1.docx]

**Supplementary Material**

**Table S1. Phase 1 diet composition (fed from 21 – 35 days-old).**

| Ingredient (%) | Control Diet | Antibiotic Diet* |
| --- | --- | --- |
| Corn  Soybean Meal  Oats  HP 300^1^  Whey  Fish meal  Plasma Protein  Fat  Lysine  Methionine  Limestone  Dicalcium Phosphate  Salt  Vitamin Premix**  Trace Mineral Mix***  Antibiotic Premix | 30.23  8.78  10.00  10.00  27.50  5.00  5.00  1.68  0.30  0.06  0.94  0.02  0.20  0.25  0.15  0 | 28.37  8.91  10.00  10.00  27.50  5.00  5.00  2.39  0.30  0.06  0.92  0.04  0.20  0.25  0.15  1.00 |
| Nutrient Content  Energy, kcal/kg ME  Crude Protein, %  Lysine, SID %  Calcium, %  Phosphorus, STTD % | 3400  23.86  1.50  0.85  0.45 | |

^1^HP 300: Hamlet Protein, Findlay, OH. * Antibiotic = Carbadox at 55 mg/kg. ** Vitamin Pre-mix supplied per kg of premix: vitamin A 4,400 IU; vitamin D 660,000 IU; vitamin E 17,600 IU; vitamin K 1,760 IU; riboflavin 3,960 mg; niacin 22,000 mg; vitamin B12 17,600 μg . *** Trace Mineral Mix supplied per kg of premix: iron 110,000 mg; copper 11,000 mg; manganese 26,400 mg; zinc 110,000 mg; iodine 198 mg; selenium 198 mg.

**Table S2. Phase 2 diet composition (fed from 36 – 49 days-old).**

| Ingredient (%) | Control Diet | Antibiotic Diet* |
| --- | --- | --- |
| Corn  Soybean Meal  Oats  HP 300^1^  Whey  Fish meal  Fat  Lysine  Methionine  Threonine  Limestone  Dicalcium Phosphate  Salt  Vitamin premix**  Trace Mineral Premix***  Antibiotic Premix | 46.82  25.18  5.00  5.00  10.00  3.00  2.28  0.30  0.10  0.05  0.89  0.73  0.25  0.25  0.15  0 | 45.90  25.25  5.00  5.00  10.00  3.00  2.63  0.30  0.10  0.05  0.89  0.73  0.25  0.25  0.15  0.50 |
| Nutrient Content  Energy, kcal/kg ME  Crude Protein, %  Lysine, SID %  Calcium, %  Phosphorus, STTD % | 3400  22.20  1.35  0.80  0.40 | |

^1^HP 300: Hamlet Protein, Findlay, OH. * Antibiotic = Carbadox at 27.5 mg/kg. ** Vitamin Pre-mix supplied per kg of premix: vitamin A 4,400 IU; vitamin D 660,000 IU; vitamin E 17,600 IU; vitamin K 1,760 IU; riboflavin 3,960 mg; niacin 22,000 mg; vitamin B12 17,600 μg . *** Trace Mineral Mix supplied per kg of premix: iron 110,000 mg; copper 11,000 mg; manganese 26,400 mg; zinc 110,000 mg; iodine 198 mg; selenium 198 mg.

**Table S3. Phase 3 diet composition (fed from 50 – 63 days-old).**

| Ingredient (%) | **Common Diet** |
| --- | --- |
| Corn  Soybean Meal | 61.51  33.84 |
| Fat | 1.24 |
| Salt  Limestone  Dicalcium Phosphate | 0.25  0.71  1.53 |
| Vitamin Premix*  Trace Mineral Premix** | 0.25  0.15 |
| L-Lysine  DL-Methionine  Threonine | 0.30  0.07  0.06 |
| Total | 100.0 |
| Calculated Analysis  Energy, kcal ME/kg  Crude Protein, %  SID Lysine, %  SID TSAA, %  Calcium, %  STTD Phosphorus  Crude fiber, % | 3350  21.82  1.23  0.69  0.70  0.35  2.54 |

* Vitamin Pre-mix supplied per kg of premix: vitamin A 4,400 IU; vitamin D 660,000 IU; vitamin E 17,600 IU; vitamin K 1,760 IU; riboflavin 3,960 mg; niacin 22,000 mg; vitamin B12 17,600 μg . ** Trace Mineral Mix supplied per kg of premix: iron 110,000 mg; copper 11,000 mg; manganese 26,400 mg; zinc 110,000 mg; iodine 198 mg; selenium 198 mg.


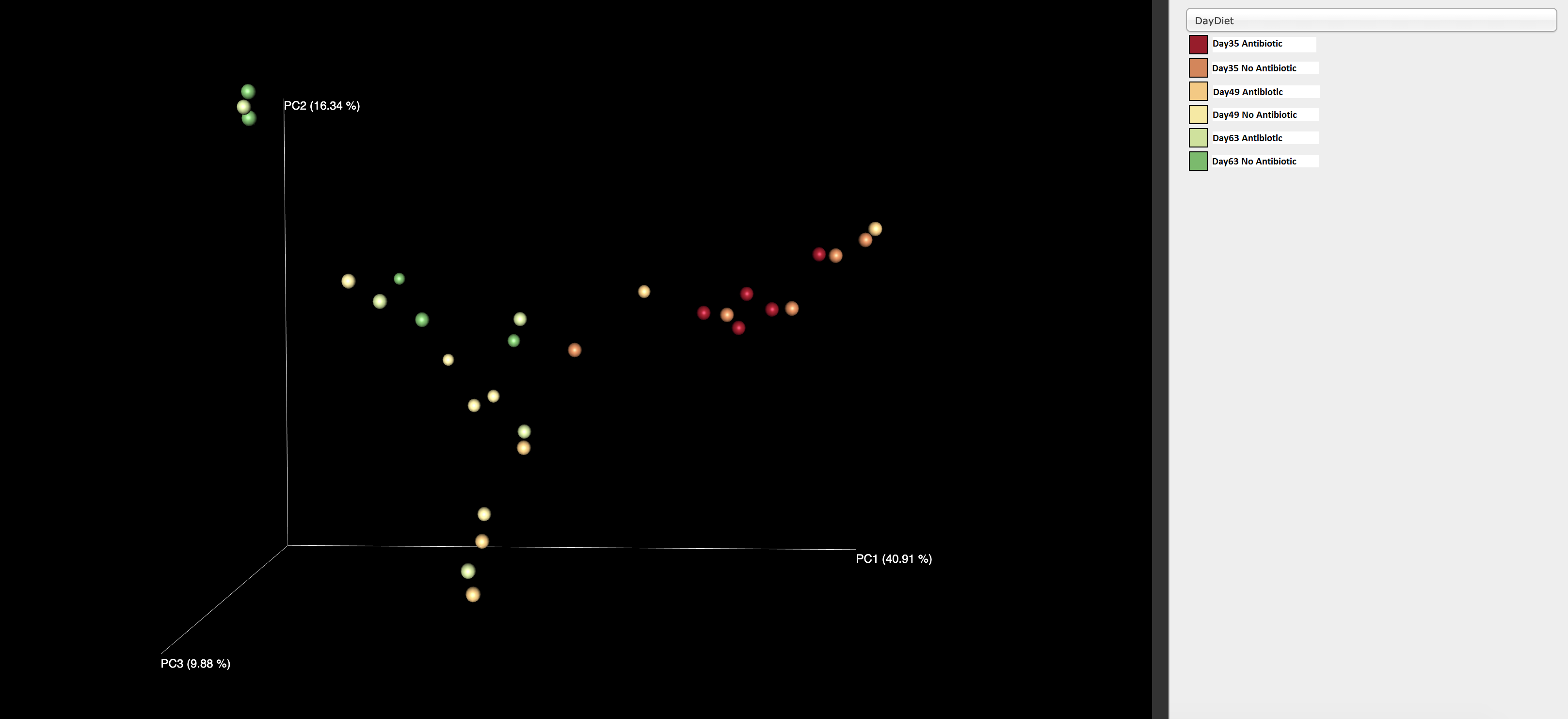


**Supplementary Figure 1.** Principal Coordinates Analysis (Euclidean Distance) showing fecal community composition of piglets in the No Antibiotic and Antibiotic groups.


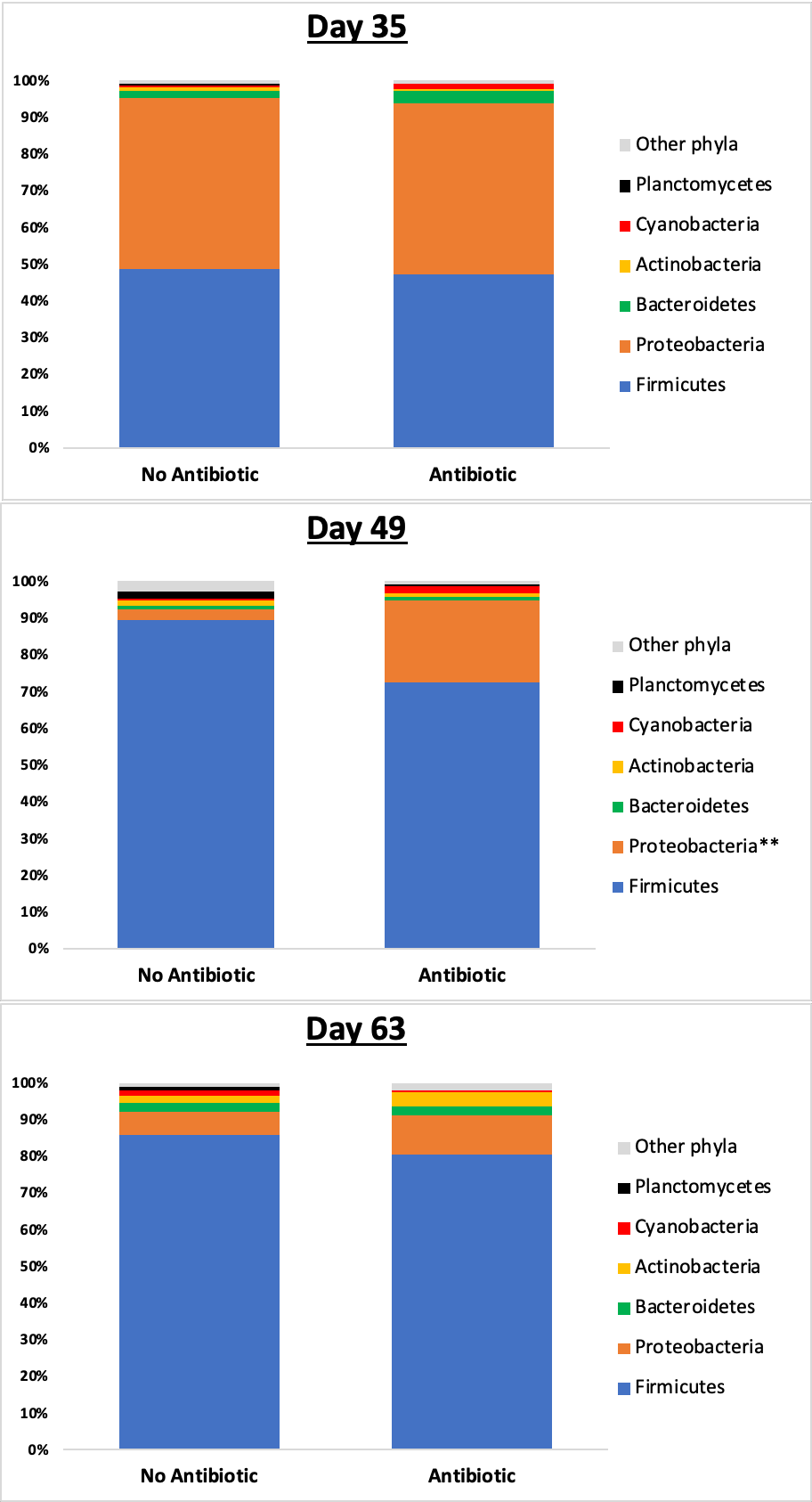


**Supplementary Figure 2.** Bacterial abundance at the phylum level in the feces of piglets in the No Antibiotic and Antibiotic groups. ** indicates a significant difference (*P* ≤ 0.05) between piglets in the two groups on each (day 35: end of phase 1; day 49: end of phase 2; day 63: end of phase 3).


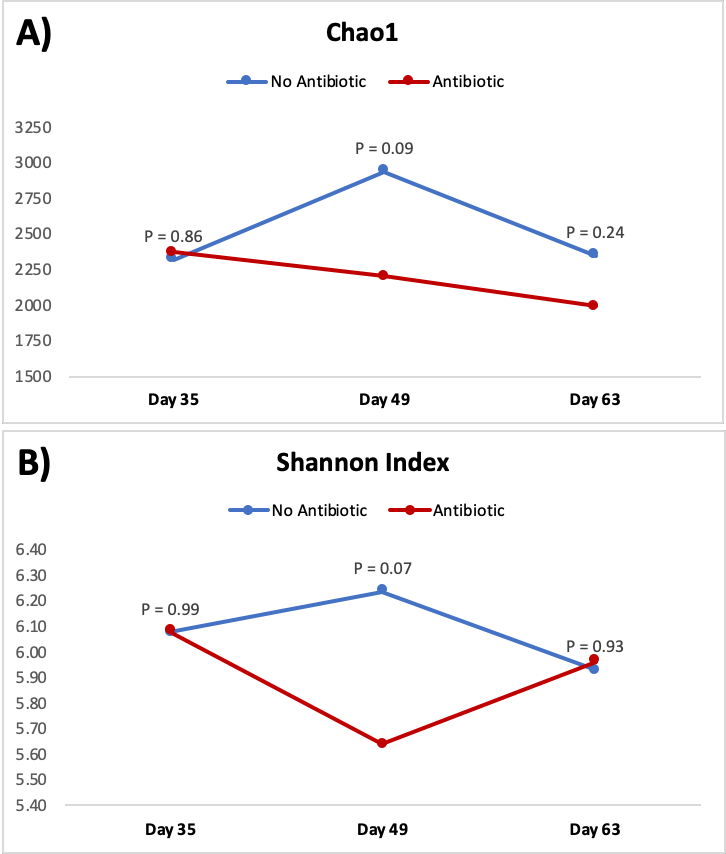


**Supplementary Figure 3.** Chao1 Index A); and Shannon Index B); calculated for piglets in the No Antibiotic and Antibiotic groups. P-values indicate the contrast between piglets in the two groups on each (day 35: end of phase 1; day 49: end of phase 2; day 63: end of phase 3).
